# Supplementary material for: DNA methylation as a contributor to dysregulation of STX6 and other frontotemporal lobar degeneration genetic risk-associated loci
Source: bioRxiv. 2025 Feb 6:2025.01.21.634065. Preprint. [Version 2] doi: 10.1101/2025.01.21.634065 (PMC11838521; doi:10.1101/2025.01.21.634065)
Supplement: Supplementary file 1 [file media-1.docx]

**DNA methylation as a contributor to dysregulation of** ***STX6*** **and other frontotemporal lobar degeneration genetic risk-associated loci**

Naiomi Rambarack^1^, Katherine Fodder^1^, Megha Murthy^2^, Christina Toomey^2,3^, Rohan de Silva^2,4^, Peter Heutink^5^, Jack Humphrey^6^, Towfique Raj^6^, Tammaryn Lashley^1^, Conceição Bettencourt^1*^

1. Department of Neurodegenerative Disease, UCL Queen Square Institute of Neurology, London, UK

2. Department of Clinical and Movement Neurosciences, UCL Queen Square Institute of Neurology, London, UK

3. The Francis Crick Institute, London, UK

4. Reta Lila Weston Institute, UCL Queen Square Institute of Neurology, London, UK 

5. German Center for Neurodegenerative Diseases, Tübingen, Germany

6. Nash Family Department of Neuroscience and Friedman Brain Institute, Icahn School of Medicine at Mount Sinai, New York, NY USA

*Corresponding Author: 
Conceição Bettencourt, PhD
Department of Neurodegenerative Disease
UCL Queen Square Institute of Neurology 

1 Wakefield Street
London WC1N 1PJ
United Kingdom

Email [c.bettencourt@ucl.ac.uk](mailto:c.bettencourt@ucl.ac.uk)

**Supplementary Figures**

 **Supplementary Fig. 1** **Analysis of DNA methylation patterns across the** ***STX6*** **locus in FTLD1m**. Note: CpGs at the promoter region showing changes in other FTLD subtypes (cg02925840 and cg05301102) were not present in this dataset due to their exclusion during data quality control pre-processing.  

FTLD1m – frontotemporal lobar degeneration DNA methylation cohort 1, TSS – transcription start site; TSS200 – 0–200 bases upstream of TSS; TSS1500 – 200-1500 bases upstream of TSS; UTR – untranslated region.

 

**Supplementary Fig. 2 Analysis of DNA methylation patterns across the** ***MAPT*** **locus in** ***GRN*** **and** ***C9orf72*** **cases from the FTLD2m cohort**. Note: No probes met the significance threshold (p<0.05) in the FTLD-TDP Type A *GRN* or Type B *C9orf72* cases compared to controls.

FTLD2m – frontotemporal lobar degeneration DNA methylation cohort 2, TSS – transcription start site; TSS200 – 0–200 bases upstream of TSS; TSS1500 – 200-1500 bases upstream of TSS; UTR – untranslated region.

**Supplementary Fig. 3 Analysis of DNA methylation patterns across the** ***GRN*** **locus in non-TDP Type A cases**. Note: No probes showed dysregulated methylation (absolute delta-beta ≥5%, p<0.05) at the promoter region in other FTLD-TDP types or FTLD-Tau cases.  

TSS – transcription start site; TSS200 – 0–200 bases upstream of TSS; TSS1500 – 200-1500 bases upstream of TSS; UTR – untranslated region.

**Supplementary Fig. 4 Analysis of DNA methylation patterns across the** ***C9orf72*** **locus in non-mutation carriers**. Note: No probes at any region showed an absolute delta-beta, i.e. mean difference when compared to controls, of ≥5%.  

TSS – transcription start site; TSS200 – 0–200 bases upstream of TSS; TSS1500 – 200-1500 bases upstream of TSS; UTR – untranslated region.
